# Multidimensional analysis reveals environmental factors that affect community dynamics of arbuscular mycorrhizal fungi in poplar roots

**DOI:** 10.3389/fpls.2022.1068527

**Published:** 2023-01-17

**Authors:** Shuo Han, Xia Wang, Yao Cheng, Guanqi Wu, Xiaoyi Dong, Xiangwei He, Guozhu Zhao

**Affiliations:** ^1^ College of Biological Sciences and Technology, Beijing Forestry University, Beijing, China; ^2^ National Engineering Research Center of Tree Breeding and Ecological Restoration, Beijing Forestry University, Beijing, China

**Keywords:** arbuscular mycorrhizal fungi(AMF), poplar, seasonal dynamics, environmental factors, diversity, glomalin

## Abstract

**Introduction:**

Poplar is a tree species with important production and application value. The symbiotic relationship between poplar and arbuscular mycorrhizal fungi (AMF) has a key role in ecosystem functioning. However, there remain questions concerning the seasonal dynamics of the AMF community in poplar roots, the relationship between AMF and the soil environment, and its ecological function.

**Method:**

Poplar roots and rhizosphere soil were sampled at the end of April and the end of October. The responses of AMF communities to season, host age, and host species were investigated; the soil environmental factors driving community changes were analyzed.

**Results:**

The diversity and species composition of the AMF community were higher in autumn than in spring. Season, host age, host species, and soil environmental factors affected the formation of the symbiotic mycorrhizal system and the AMF community. Differences in the communities could be explained by soil pH, total nitrogen, total phosphorus, total potassium, available potassium, and glomalin content.

**Discussion:**

The AMF community was sensitive to changes in soil physicochemical properties caused by seasonal dynamics, particularly total potassium. The change in the mycorrhizal symbiotic system was closely related to the growth and development of poplar trees.

## Introduction

Arbuscular mycorrhizal fungi (AMF) are important functional components of soil ecosystems. They are widely distributed, form a symbiotic relationship with the roots of most higher plants, and have an important role in ecosystem regulation. Nutrient exchange between AMF and plant roots facilitates host absorption and the recycling of mineral elements. In addition to improving the host plant’s ability to absorb water and nutrients, AMF can enhance plant resistance to abiotic stress, enhance the stability of soil aggregates, and stabilize and improve soil structure.

In recent years, there has been increasing attention toward seasonal changes in the AMF community and their interactions with plants. The seasonal dynamics of biological and non-biological factors strongly affect AMF communities (Reyes et al., 2019). The abundance and uniformity of AMF spores in the root system of *Alsophila spinulosa* were higher in the rainy season, but the degree of colonization was higher in the dry season (Lara-Pérez et al., 2014). The AMF community diversity in the rhizosphere soil of *Juglans mandshurica* was greater in July than in May or September; nitrate nitrogen and water-soluble phenol contents were the main factors driving the seasonal change (Ji et al., 2021). In drought experiments, the hypha density, spore density, and root colonization rate of AMF were significantly higher in summer than in winter; the community compositions of soil and root AMF were significantly affected by the seasons (Maitra et al., 2019). There were significant differences in AMF community composition among soil and root systems in three maize growth stages, possibly because of the effects of fertilization and irrigation (Lü et al., 2020). Sporulation and community composition of AMF depend on the host (Bever et al., 1996). For example, in Inner Mongolian grassland and semiarid Mediterranean prairies, mycorrhizal colonization differed among plant species (Su et al., 2011; Torrecillas et al., 2012). In natural ecosystems, AMF host preference plays an important role in maintaining plant diversity. AMF colonization in some medicinal plants in Chittagong, Bangladesh, changed due to the effects of climate, soil, host relationship, and species diversity (Halder et al., 2015). Greater knowledge concerning the effects of specific seasons on AMF community composition in a forest ecosystem can help to understand their role in plant growth and development.

Because of its high growth rate, poplar is widely used in various industrial sectors, as well as in the reforestation of post-agricultural land, replanting of areas degraded by human activities, production of bioenergy (Sebastiani et al., 2004; Gottel et al., 2011; Langer et al., 2012), and rehabilitation programs along riverbanks (Smulders et al., 2008; Szuba, 2015). Mycorrhizal colonization of poplar can provide mineral nutrition and improve its resistance to stress, this symbiotic relationship plays an important role in ecosystem function. Poplar can form a tripartite symbiosis with ectomycorrhiza (ECM) and AMF (Gonçalves and Martins-Loução, 1996; Karliński et al., 2010). The ECM dominates more mature trees, while AMF colonizes poplar throughout its entire growing period, especially in the seedling stage (Aguillon and Garbaye, 1990; Quoreshi and Khasa, 2008). The mechanism of mycorrhizal replacement is related to the change of soil nutrients (Read, 1991), although the symbiotic relationship between AMF and poplar still needs to be studied. The symbiosis of poplar with AMF can improve its growth in heavy metal-contaminated soil (Cicatelli et al., 2014). Moreover, AMF can remediate soil contamination caused by high zinc concentrations in *Populus alba* and *Populus nigra*; mycorrhiza can increase poplar biomass. AMF can also increase nitrogen fixation in poplar roots, retain soil nitrogen, reduce soil nitrogen leaching, reduce the risk of environmental pollution (Fang et al., 2020), and improve water absorption and utilization efficiency under drought stress (Liu et al., 2015).

Seasonal changes often lead to changes in temperature and soil conditions, and litter from plant growth can transfer soil nutrients. Soil microbes are highly reactive to soil nutrients and seasonal changes in soil climate (Rasche et al., 2011); these changes have substantial effects on the diversity and composition of soil bacterial and fungal functional genes (Siles and Margesin, 2017). Soil environmental heterogeneity in different seasons greatly affects the spatiotemporal dynamics of microbial communities. Despite the obvious importance of AMF for nutrient uptake and stress resistance during poplar growth, the seasonal dynamics of the AMF community in poplar remain unclear. The elucidation of poplar AMF community composition in an undisturbed environment will benefit future bioremediation planning. As a secretion product of AMF hyphae, glomalin-related soil protein (GRSP) is an important source of soil organic carbon pools (Kumar et al., 2018). Previous studies have suggested a significant positive correlation between organic carbon, nitrogen, and GRSP content in soil (Wright and Anderson, 2000; Wright and Nichols, 2002). The content and distribution of GRSP in soil is affected by AMF and farmland management practices such as host plants, soil type, fertilization, and tillage (Wang et al., 2016). It is necessary to investigate the variation of GRSP content under different factors.

The aim of this study was to assess the effects of soil parameters changes caused by seasonal dynamics, host age and species on AMF community and soil glomalin. We hypothesized that: (i) the colonization relationship between AMF and poplar is more close in autumn, and the difference of host age and species will also affect soil parameters; (ii) the species richness and diversity of AMF community will increase in autumn; (iii) soil glomalin content will increase due to the high richness of AMF community, further affecting soil carbon pool. We investigated soil environmental factors and root AMF communities with respect to season, tree age, and tree species. We examined soil physical and chemical properties, root colonization rates, soil glomalin-related protein contents, and spore density. Their effects on AMF community diversity and structure were investigated based on Illumina amplicon sequencing. We then constructed a structural equation model to explore the interactions among soil parameters, AMF community diversity, and glomalin content. This study explored the association mechanism of the soil-AMF community, to provide insights into the ecological service function of AMF in forest, ecosystems and a theoretical basis for understanding the role of mycorrhiza coping with climate change. The study results will also serve as a reference for appropriate use of AMF in afforestation and the development of mycorrhizal biotechnology.

## Materials and methods

### Sample collection

Poplar samples were collected from a research site in Guanxian County, Shandong Province, China (115°22’12” E, 36°32’24” N, 40 m altitude), which is located in a warm temperate monsoon zone with a continental semi-arid climate, four distinct seasons, and sufficient sunshine. The mean annual temperature precipitation, and air pressure are 13.1°C, 576.4 mm, and 101.2 kPa, respectively. In April (spring) and October (autumn) 2021, we collected 0-20-cm rhizosphere soil samples of young, adult, 30-year-old, and 100-year-old *Populus tomentosa*, and young and adult *P. nigra* and *Populus simonii* in spring and autumn in the forest area; young roots were also excavated. At each sampling site, there were five sampling points: one at the center of the sampling area and four at the midpoints between the center and four corners. For sampling, the center point was set 0.5 m from a tree trunk. The five soil samples from each sampling location were mixed to yield one sample; this was repeated three times for each sample location (He et al., 2021). All samples were sealed in an icebox and transported to the laboratory. The root samples were rinsed with clean water and the surface moisture was removed, and stored at −20°C. The rhizosphere soil samples were stored at 4°C; soil subsamples were air-dried for analysis of physicochemical properties.

### Soil analyses

The pH was measured with a pH meter at a water/soil mass ratio of 2.5:1. Electrical conductance (EC) was measured under a water/soil mass ratio of 5:1. Soil organic matter (SOM) was determined by potassium dichromate titration. Soil total nitrogen (N) was determined by the Kjeldahl method; ammonium nitrogen (NH_4_^+^·N) and nitrate nitrogen (NO_3_^−^·N) were determined by ultraviolet spectrophotometry. Total phosphorus (P) and available phosphorus (AP) in soil were extracted by perchloric acid–sulfuric acid and sodium bicarbonate, respectively; they were determined by the molybdenum–antimony colorimetric method. Total potassium (K) in soil was determined by flame atomic absorption spectrophotometry. Soil available potassium (AK) was determined by inductively coupled plasma atomic emission spectroscopy (Bao, 2018).

### Colonization intensity of AMF

In accordance with the method established by Phillips (Phillips and Hayman, 1970), poplar root samples stored in FAA fixative were transferred to a 10% potassium hydroxide solution and heated in a 90°C water bath for 30–60 min. After they had softened, root samples were soaked in 2% HCl for 5–10 min, then stained with Trypan blue. For each sample, 30 root fragments were selected and cut into 1-cm root segments. Ten segments were placed onto a glass slide in a sequential manner, then treated with lactic glycerol for tableting; three slides were made for each sample. Photographs were observed under a light microscope, and the colonization intensity was measured with the MI method (Mcgonigle et al., 1990).

### AMF spore density in soil

The collected soil samples were mixed evenly, and 10-g subsamples were weighed to determine the spore content. AMF spores were centrifuged by modified wet sieving and the sucrose centrifugation method (Liu and Chen, 2007). After centrifugation, the 60% sucrose solution was poured directly onto filter paper for filtration; fresh viable spores were counted by observing the filter paper under a microscope, and the number of AMF spores per unit weight of soil was determined.

AMF spore density (number/g) = number of spores in 10 g soil/10 g soil

### Detection of soil glomalin-related soil protein

The glomalin-related soil protein (GRSP) was extracted using a modified version of the method proposed by Wright et al. (Wright and Upadhyaya, 1998). The method for extraction of easy-to-extract glomalin-related soil protein (EEG) was as follows: 0.5 g of air-dried soil was weighed into a centrifuge tube, and 4 mL of 20 mmol/L sodium citrate extract (pH = 7.0) was added. The sample was extracted at 103 kPa and 121°C for 30 min, depressurized, and centrifuged at 4000 rpm for 6 min; the supernatant was collected for analysis. The method for extraction of total glomalin-related soil protein (TG) was as follows: 0.1 g of air-dried soil was weighed into a centrifuge tube, and 4 mL of 50 mmol/L sodium citrate leaching solution (pH = 8.0) was added. The sample was extracted at 103 kPa and 121°C for 60 min, depressurized, and centrifuged at 4°C and 4000 rpm for 6 min. The supernatant was collected, fresh leaching solution was added, and the extraction was repeated at least twice until the extracted supernatant no longer showed the typical GRSP yellow-brown color. Finally, the total collected supernatant was combined and thoroughly mixed for analysis.

The protein content was determined by the Coomassie brilliant blue method: 0.5 mL of the solution to be measured was collected and mixed with 5 mL of Coomassie brilliant blue G-250 dye. The absorbance was recorded at 595 nm by a microplate reader. Using bovine serum albumin as the standard substance, the standard curve was established by the same method. Finally, the protein concentration and GRSP content in the solution were calculated from the standard curve, and the results are expressed as mg of protein in 1 g of soil (Rillig, 2004).

### DNA extraction and amplicon sequencing

DNA was extracted from poplar root samples with three replicates for each set of samples. The DNA concentration and purification were measured using a NanoDrop 2000 ultraviolet-visible spectrophotometer. The 18S rRNA gene of AMF was amplified by nested polymerase chain reaction (PCR). In the first round of PCR, the forward and reverse primers AML1 (5’-ATCAACTTTCGATGGTAGGATAGA-3’) and AML2 (5’-GAACCAACTTTTTTTTTTCC-3’) were used (Lee et al., 2008); in the second round, AMV4.5NF (5’-AAGCTCGTAGTTTTTCG-3’) and AMDGR (5’-CCCAACTATCCCTATTAATCAT-3’) were used (Van Geel et al., 2014). The PCR procedure was as follows: denaturation at 95°C for 3 min; 30 cycles of 95°C for 30 s, 55°C for 30 s, and 72°C for 45 s; and a final extension for 10 min at 72°C. The second round of PCR was identical to the first, except the number of cycles was changed to 35. Sequencing was carried out using the NovaSeq PE250 platform (Illumina, San Diego, CA, USA). Sequence data from non-target sequences were removed, then sequences were classified using USEARCH software. The dereplicated data were classified into different amplicon sequence variants (ASVs). ASVs were identified from reference sequences in the MaarjAM library (Öpik et al., 2009; Lekberg et al., 2018), and the community composition of each sample was counted at the genus and species taxonomic levels. Raw sequencing data was submitted to the NCBI Sequence Read Archive and is available under Bioproject PRJNA880710.

### Statistical analyses

The Chao1 and observed species indexes were used to characterize richness, Shannon and Simpson indexes were used to characterize diversity, Faith’s PD index was used to characterize evolution-based diversity, Pielou’s evenness was used to characterize uniformity, and Good’s coverage index was used to characterize coverage. For the ASV tables, the mean score was used as the alpha diversity index when the maximum leveling depth was chosen in QIIME2. Data processing and statistical analysis were carried out in R (v4.1.2). The LeveneTest and qqplot functions were used to determine whether the data conformed to a normal distribution, and the Anova function was used to perform variance analysis. The lm function was used to calculate the linear correlations between variables. The ggplot2 package (v3.3.5) (Wickham, 2016) was used for plotting. Pearson’s correlation coefficient was used to calculate correlations between environmental factors. Spearman’s correlation coefficient and p-values between environmental factors and AMF community diversity were analyzed using the corr.text function in the psych package (v2.1.9) (Revelle, 2017); the p-values were adjusted using the false discovery rate method.

For the ecological ranking of AMF community composition and environmental factors, the decorana function in the vegan package (v2.5.7) (Oksanen et al., 2021) was used for method selection. Furthermore, the cca function was used to analyze the unimodal model of ASV level, and the rda function was used to analyze the linear model of diversity index. To test the significance of the ranking analysis, permutational multivariate analysis of variance (PERMANOVA) and analysis of similarities (ANOSIM) were implemented by the adonis and anosim functions, respectively. Distance-based redundancy analysis (db-rda, based on Bray–Curtis dissimilarity) was calculated by the RDA function of the ggvegan package (v0.1.0) (Simpson, 2015) and used to examine the extent to which environmental factors explained AMF richness and diversity; it was also used to calculate changes in community composition. Canonical correspondence analysis was used to calculate the relative distances between ASV level and environmental factors. The significance of the ranking was analyzed by the anova.cca function (step = 1000).

The randomForest package (v.4.6.14) (Liaw and Wiener, 2002) was used to quantify the relative contributions of season, tree age, tree species, and environmental factors to AMF richness and diversity predictions. Based on the correlation analysis and the random forest prediction results, a structural equation model was constructed using the sem function of the lavaan package (v0.6.9) (Rosseel, 2012). The model was improved by the modificationindices function; model fitting indices were used for evaluation, including chi-squared (χ^2^)/d < 2, P > 0.05, root mean square error of approximation (< 0.05), goodness of fit index (> 0. 99), and comparative fit index (> 0.95) (Hooper et al., 2008).

## Results

### Environmental factors affecting poplar rhizosphere soil

**Figure 1:**
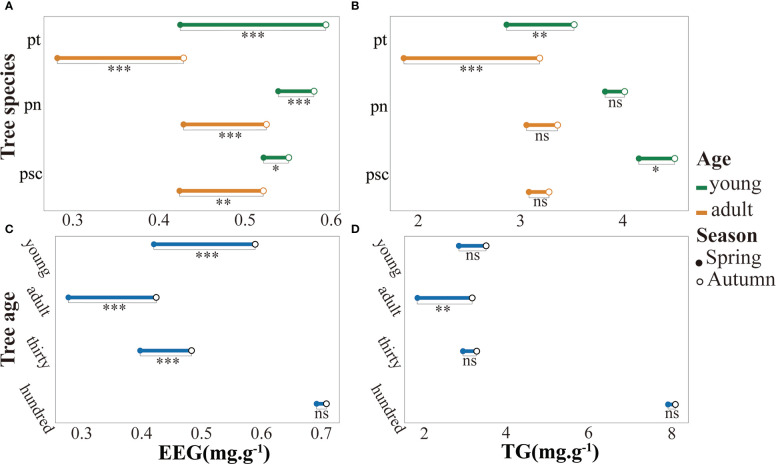
Glomalin-related soil protein (GRSP) contents in poplar rhizosphere soil. a. The EEG contents in *Populus tomentosa*, *Populus nigra*, and *Populus simonii.* b. The TG contents in *P. tomentosa*, *P. nigra*, and *P. simonii*. c. The EEG contents in rhizosphere soil of young, adult, 30-year-old, and 100-year-old *P. tomentosa*. d. The TG contents in rhizosphere soil of young, adult, 30-year-old, and 100-year-old *P. tomentosa*. EEG, easy-to-extract glomalin-related soil protein, TG, total glomalin-related soil protein; pt, *P. tomentosa*, pn, *P. nigra*; psc, *P. simonii*. Comparisons were made using Student's t-test. ns, not significant; *0.01 < *p* < 0.05; **0.001; < *p* < 0.01; ***p < 0.001.

**Figure 2:**
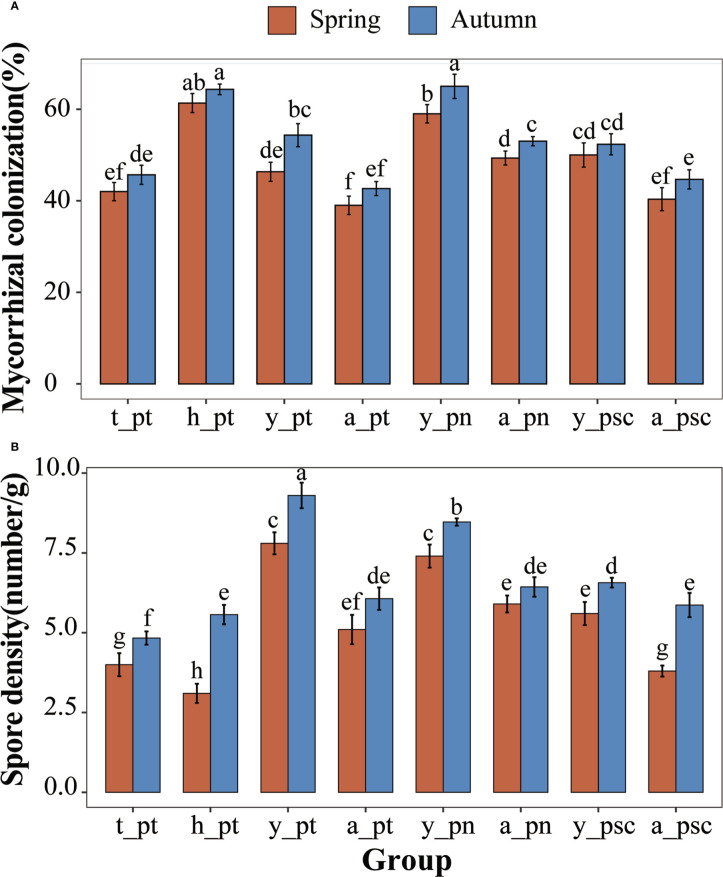
AMF colonization of poplars. **(A)** AMF colonization of poplar. **(B)** Spore density in rhizosphere soil of poplars. The lowercase letters y, a, t, and h, indicate young, adult, 30-year-old, and 100-year-old, respectively. Different letters on top of the bars indicate significant differences (p < 0.05). All data are presented as means ± standard errors.

In autumn, the pH value and K of poplar rhizosphere soil were higher, whereas the NH_4_^+^·N content was considerably lower (**Table S1**). Seasonal factors and tree age factors have strong effects on soil physical and chemical properties (**Table S2**). The EEG and TG contents in rhizosphere soil of *P. tomentosa* were significantly increased in autumn (**Figure 1**). Furthermore, among the three tree species, the GRSP content of young poplar was considerably higher than the GRSP content of adult poplar (**Figures 1A, B**). The GRSP content of 100-year-old *P. tomentosa* was much higher than the GRSP content of other samples, and it did not substantially change between the two seasons. The GRSP content of young *P. tomentosa* was higher than the GRSP contents of adult and 30-year-old trees (**Figures 1C, D**). The stability of soil aggregates varied according to poplar growth. Analysis of variance showed that different tree ages, tree species, and seasons had significant effects on EEG content in poplar rhizosphere soil. The TG content varied according to tree age and tree species, but it was not strongly affected by season (**Table S3**). Tukey HSD analysis of GRSP content in soil further showed that the difference of age was reflected in 100-year-old, and the difference of tree species was reflected in *P. tomentosa* (**Figure S1**).All poplars were colonized by AMF. Poplar roots were infected by AMF hyphae with obvious vesicle structure (**Figure S2A**), and abundant spores were observed in the rhizosphere soil (**Figure S2B**). Analysis of variance showed that different tree ages and tree species had significant effects on AMF colonization intensity, whereas seasonal factors had no significant effects (**Table S3**). Tukey HSD analysis of AMF colonization density showed that the difference in age was reflected in 100-year-old, and the difference in species was reflected in *P. nigra* (**Figure S1**). The AMF colonization intensity and spore density in rhizosphere soil were significantly higher in autumn than in spring (**Figure 2**). 100-year-old *P. tomentosa* had the highest AMF colonization intensity, followed by young *P. tomentosa*. The colonization intensity of *P. nigra* was highest; it was higher in young poplar than in adult poplar (**Figure 2A**). Seasonal factors, tree age, and tree species influenced spore density (**Table S3**). Tukey HSD analysis of spore density showed that the difference in spore density caused by age was reflected in the youth, and the difference in species was reflected in *P. simonii* (**Figure S1**). The spore density of *P. tomentosa* significantly decreased with increasing tree age. The spore density of *P. tomentosa* was highest in autumn, followed by *P. nigra* (**Figure 2B**).

### AMF community of poplar root system

**Figure 3:**
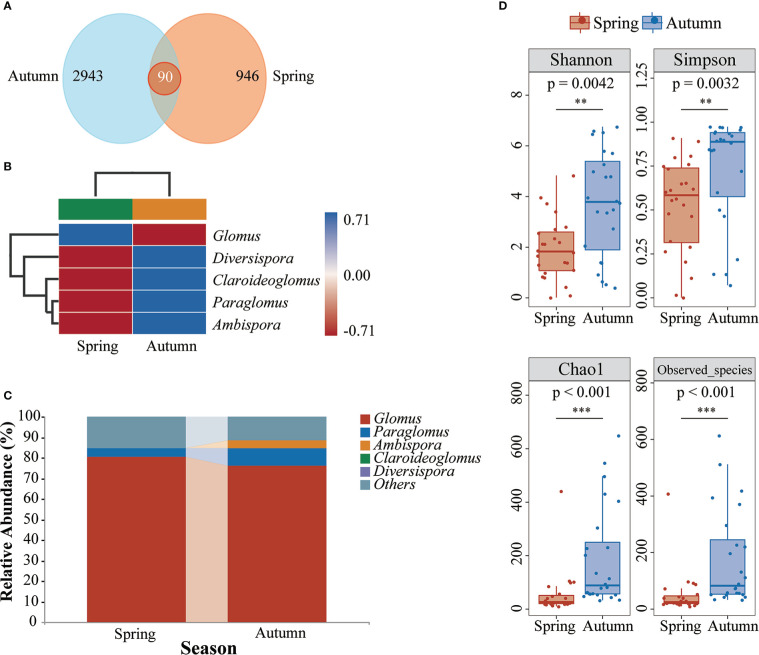
Effects of seasonal changes on the AMF community composition of poplar roots. **(A)** Venn diagram of AMF communities based on the ASV level. **(B)** Relative abundances of species at the genus level. **(C)** Heatmap of species composition at the genus level. **(D)** Alpha diversity indices of the AMF communities of poplar roots. **0.001 < *p* < 0.01; ***p < 0.001.

In total, 3,726,065 valid readings were obtained after removal of singleton. After standardization, 3,979 AMF ASVs were detected in poplar rhizosphere soil, including 1,036 in spring and 3,033 in autumn. There were 90 ASVs common to both seasons, 946 ASVs unique to spring, and 2,943 ASVs unique to autumn (**Figure 3A**). Among the different tree ages of *P. tomentosa*, the unique ASVs of young trees were highest, followed by 100-year-old trees (**Figure S3A**). Among the three poplar species, *P. nigra* in autumn had the largest number of ASVs (**Figure S3B**). All ASVs were identified at the genus level. The dominant genus of AMF in poplar roots was *Glomus*. The relative abundances of *Paraglomus*, *Diversispora*, *Claroideoglomus*, and *Ambispora* increased in autumn (**Figures 3B, C**). Cluster analysis heatmaps showed that the AMF community compositions among tree ages and species differed between spring and autumn (**Figure S4**). The number of high-abundance AMF genera increased in autumn, indicating that the different seasons had distinct selectivity for AMF in poplar roots.

### Diversity of AMF in poplar roots

AMF richness and alpha diversity were significantly higher in autumn than in spring (**Figure 3D**). The community diversity was not affected by season or tree age (**Figure S5A**). There were differences in root AMF diversity among tree species, with the highest level in *P. nigra* in autumn (**Figure S5B**).

Based on the ASV level, the Bray–Curtis distance matrix was used for non-metric multidimensional scaling and principal component analysis. In spring and autumn, the cumulative explanatory power of AMF component analysis under four tree ages of *P. tomentosa* reached 31.7% (**Figure S6A**), and the cumulative explanatory power of tree species differences among *P. tomentosa*, *P. nigra* and *P. simonii* was 24.6% (**Figure S6B**). Spring and autumn samples showed distinct grouping patterns. The root AMF community significantly varied with tree age in both seasons (**Figures S6A, C**). The AMF communities of the three poplar species were distinct, but the distances between tree species were similar (**Figures S6B, D**).

### Correlation regression analysis of environmental factors

**Figure 4:**
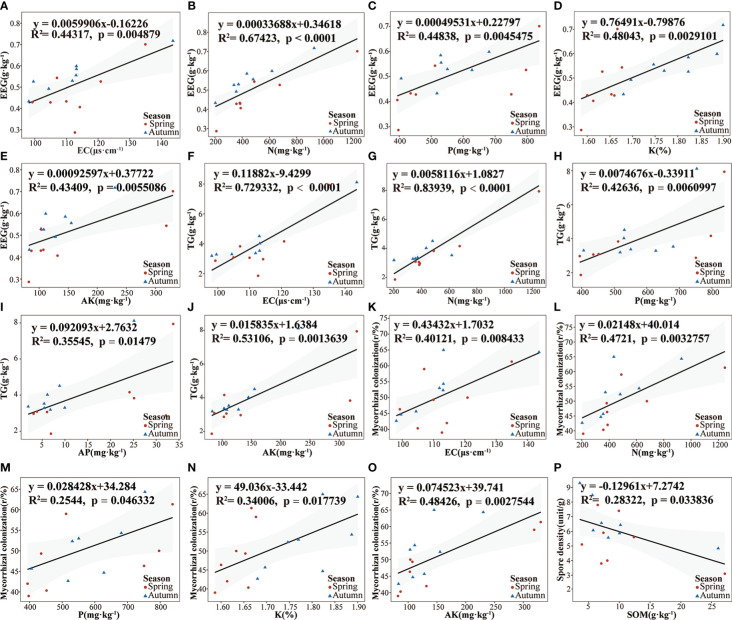
Linear regression relationships between soil physical and chemical properties and AMF-related environmental factors. **(A–E)**. EEG; **(F–J)**. TG; **(K–O)**. Mycorrhizal colonization; **(P)**. Soil spore density. CR, mycorrhizal colonization rate, SD, spore density; All linear regressions were fitted using the OLS model; gray shading represents 95% confidence intervals.

Linear correlation analysis was used to examine the relationships among EEG, TG content, spore density content, root AMF colonization intensity, and soil physical and chemical properties of poplar rhizosphere soil. The EEG content in rhizosphere soil was significantly positively correlated with EC, N, P, K, and AK (**Figures 4A–E**). TG content was positively correlated with AP, P, AK, and N; it was significantly positively with EC (**Figures 4F–J**). The colonization intensity of AMF was positively correlated with soil P and K; it was significantly positively correlated with EC, N, and AK (**Figures 4K–O**). Spore density was negatively correlated with SOM (**Figure 4P**). Pearson correlation coefficients among environmental factors were consistent with the above results. There were significantly positive correlations among EEG content, TG content, and root colonization intensity (**Figure S7**).

Principal component analysis was performed on the environmental factors associated with poplar rhizosphere soil. The environmental factors explained 73.45% (**Figure S8A**) of the age differences of *P. tomentosa* between the two seasons. For different tree species, the overall interpretation rate was 60.94% (**Figure S8B**), with significant segregation between spring and autumn samples.

### AMF community response and its potential drivers

**Figure 5:**
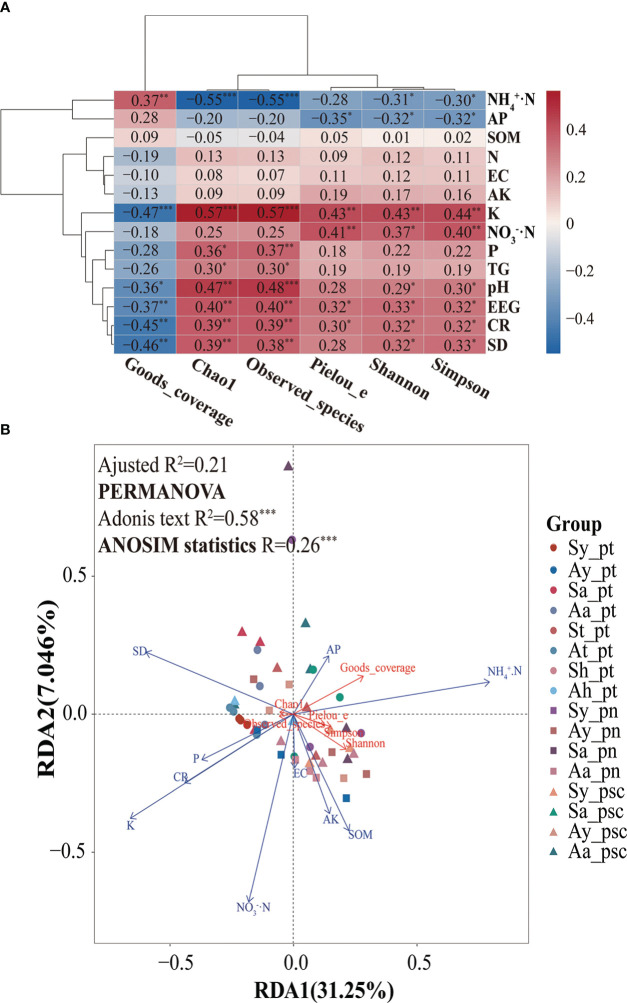
AMF community response and potential drivers. **(A)** Heatmap of the correlation analysis between the alpha diversity of poplar root AMF communities and environmental factors. **(B)** Constrained ordination analysis (db-RDA) of the alpha diversity and environmental factors of the AMF community in poplar roots. *0.01 < *p* < 0.05; **0.001 < *p* < 0.01; ***p < 0.001.

**Figure 6:**
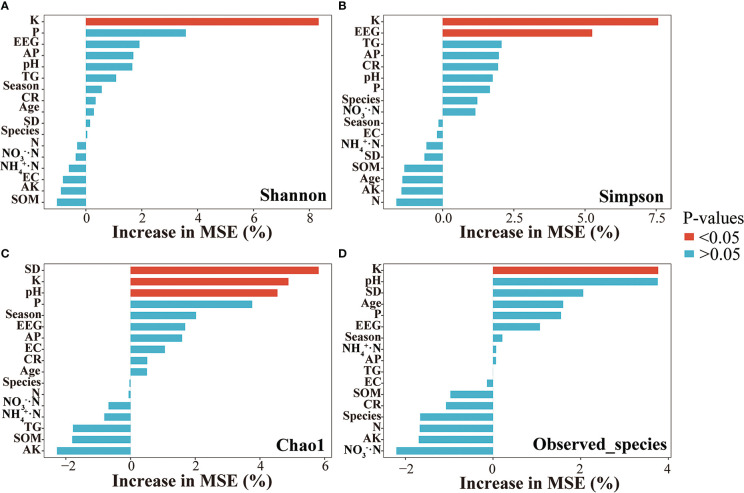
Random forest analysis of the alpha diversity of poplar root AMF communities. **(A)** Shannon Index. **(B)** Simpson Index. **(C)** Chao1. **(D)** Observed species.

In this study, the alpha diversity indices of AMF community were further explored. The Spearman correlation heatmaps of alpha diversity indices and environmental factors showed that the soil AMF community richness was positively correlated with soil K, P, and pH; it was negatively correlated with soil NH_4_^+^·N. Moreover, the increase in community richness was associated with increases in soil EEG and TG contents, root colonization intensity, and spore density (**Figure 5A**). The diversity of the AMF community in soil was positively correlated with K, NO_3_^−^·N, and pH; it was negatively correlated with NH_4_^+^·N and AP. The community evenness of soil AMF had a significantly positive correlation with K and NO_3_^−^·N; it had a negative correlation with AP. Sequencing coverage was positively correlated with soil NH_4_^+^·N but negatively correlated with K and pH. Furthermore, according to constrained ordination analysis (db-RDA, adjusted R^2^ = 0.21), these environmental factors associated with poplar roots explained 38.3% of poplar AMF community diversity and richness; axis 1 explained 31.25% of the total variation and axis 2 explained 7.05% of the total variation. Among all environmental variables, soil K had the greatest impact on AMF richness and diversity (**Figure 5B**). Random forest analysis showed that soil K could predict the differential responses of AMF richness and diversity of poplar roots, whereas soil EEG content could predict community diversity, and pH and spore density could predict community richness (**Figure 6**).

### Soil environmental factors that may regulate the AMF community

**Figure 7:**
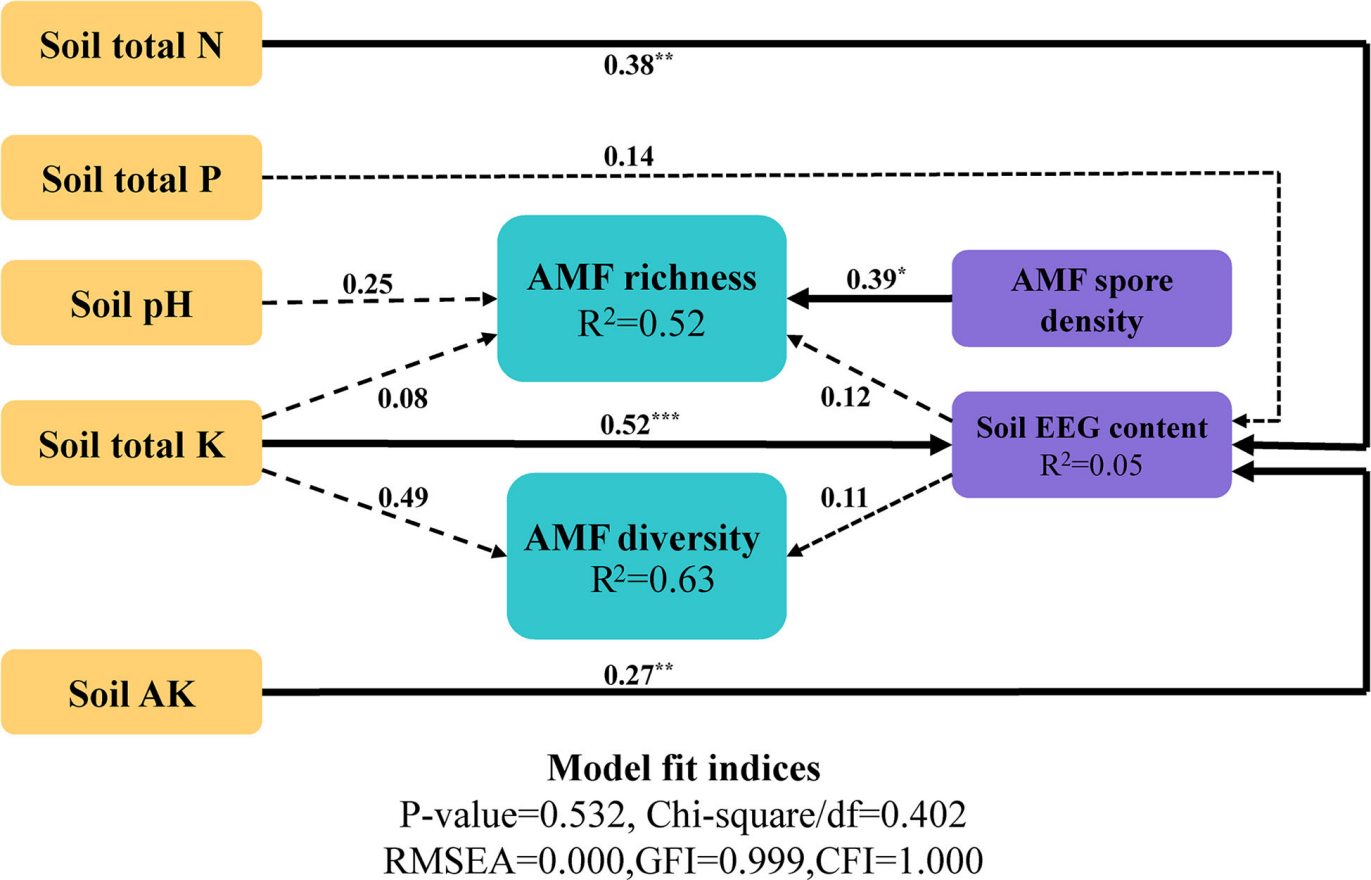
Structural equation model of the effects of environmental factors on the AMF community. Significant pathways are marked with asterisks *0.01 < *p* < 0.05; **0.001 < *p* < 0.01; ***p < 0.001.

After identification of the variables and comparison of the models, the optimal model was determined. The structural equation model was closely matched to the data: comparative fit index = 1.000; root mean square error of approximation = 0.000 (**Figure 7**). In the optimal model, the Simpson index was used to represent species diversity, Chao1 was used to represent species richness, and soil EEG content was used to represent AMF-related environmental factors; soil environmental factors were added into the model as independent variables. The results of the model showed that soil N, K, and AK had significantly positive effects on EEG content, with path coefficients of 0.38, 0.52, and 0.27, respectively. The model explained 52% of the richness and 63% of the diversity of the AMF community. Soil environmental factors had distinct direct and indirect effects on AMF species diversity and richness; AMF spore density in soil had significantly positive effects on community richness. In summary, soil environmental factors played important roles in regulating the AMF community composition and EEG content, with direct and indirect regulatory effects.

## Discussion

Most studies of AMF have been carried out in Petri dishes, greenhouses, and farmland; there have been few studies in natural ecosystems (Ohsowski et al., 2014). In this study, dynamic changes in the rhizosphere environment and root AMF community of poplar were investigated in two seasons. The stable colonization of AMF in roots and spores in soil showed that poplar formed a strong symbiotic relationship with AMF. In forest ecosystems, AMF can protect poplars from plant diseases and insect pests (Jiang et al., 2021); the inoculation of appropriate AMF can be used for afforestation and restoration of contaminated land (Bojarczuk et al., 2014). The application of AMF in poplar bioremediation is promising (Plouznikoff et al., 2016). We investigated the root AMF composition of poplar, then explored the root AMF community response during seasonal changes in different tree ages and species, and finally analyzed the effects of environmental factors on the AMF community.

The soil environment is an important determinant of the growth of mutually beneficial soil users with distinct successional sequences (Sikes et al., 2014). The soil carbon fixation ability of AMF was highest in the rainy season (Zhang et al., 2016).The physical and chemical properties of poplar rhizosphere soil changed according to the season. Seasonal differences in GRSP content can indicate the AMF activity in the plant rhizosphere. In previous studies, the GRSP level was higher in the rainy season than in the dry season (Reyes et al., 2019; Silva et al., 2020). In this study, the soil EEG content was significantly higher in autumn. However, the TG content, which is more stable in terms of soil ecology, did not significantly change. The EEG content may be associated with different AMF species during colonization because of changes in AMF community composition based on seasonal dynamics. The density of AMF spores in soil was significantly higher in autumn. However, there was no significant difference in root colonization intensity. Temperature is an important factor that affects AMF community changes (Cotton, 2018; Rasmussen et al., 2018; Davison et al., 2021). The AMF community significantly changed according to the season; the richness and diversity of the AMF community were higher in autumn. The AMF community was closely related to changes of environmental factors under seasonal dynamics. AMF resources were abundant in poplar roots and the number of detected ASVs was higher in autumn. In the AMF community of poplar roots, *Glomus* remained the dominant genus in both seasons. Previous studies have also shown that *Glomus* has a broad global distribution, is a dominant genus in many forests (Öpik et al., 2006; Jamiołkowska et al., 2018), and extensively colonizes woody plants such as coconut trees (Lara-Pérez et al., 2020) and araucaria (Vilcatoma-Medina et al., 2018). However, the AMV4.5NF/AMDGR primer pair used in this study preferentially amplified Glomeraceae sequences at the expense of Ambisporaceae, Claroideoglomeraceae, and Paraglomeraceae sequences; it also sometimes produced a large number of non-Glomeromycota sequences (Van Geel et al., 2014). These preferential amplification characteristics may have led to some test bias.

The main soil nutrient factors significantly varied according to tree age. P and N affect the AMF community composition (Chen et al., 2014; Van Geel et al., 2015). In this study, the contents of P, N, SOM, and K were much higher in the rhizosphere soil of 100-year-old *P. tomentosa* than in the rhizosphere soil of other trees. Tree age also had significant effects on GRSP, mycorrhizal colonization intensity, and spore density. The GRSP level was significantly higher in 100-year-old *P. tomentosa* than in other ages of *P. tomentosa*. Among the three poplar species, the GRSP level of young poplar was significantly higher than the GRSP level of adult poplar. This result is consistent with the findings in previous studies, which showed that the spore density of young olive rhizosphere was significantly higher than the spore density of old plantations (Meddad-Hamza et al., 2017). Our study found that the colonization intensity of mycorrhiza was consistent with the tendency of GRSP in rhizosphere soil to change according to tree age. This may be because the nutrients of mature trees return to the soil in the form of litter, which explains why the soil is high in potassium. In young poplars, the AMF community of the roots helps the tree to absorb nutrients necessary for growth. Furthermore, the colonization intensity of AMF was higher in old grape roots than in young grape roots (Vukicevich et al., 2019). A previous survey on an island polluted with volcanic ash showed that AMF colonization in plant roots was positively correlated with the GRSP concentration in rhizosphere soil (Bedini et al., 2010). There is evidence that the AMF species composition of older trees is more diverse than the AMF species composition of younger trees (Hart et al., 2014). These differences in community composition were also found in our investigation. There was a large number of unidentified AMF resources in young *P. tomentosa*, possibly because of the lack of sequences in the MaarjAM database. Notably, the abundances of *Paraglomus* and *Ambispora* were high in 30-year-old *P. tomentosa.*

This study revealed highly variable AMF communities in *P. tomentosa*, *P. nigra*, and *P. simonii*; it demonstrated some differences in the mean AMF richness and diversity. There were significant differences in soil pH, SOM, NO_3_^−^·N, AK, GRSP, mycorrhizal colonization intensity, and spore density according to tree species. A previous investigation of mycorrhiza subjected to Zn stress showed that the AMF colonization intensity of *P. nigra* was higher than the AMF colonization intensity of *P. tomentosa* (Lingua et al., 2008). Our study also showed that *P. nigra* root mycorrhizal colonization intensity was high, and its rhizosphere soil had the highest GRSP content. The spore density of *P. simonii* was lower than the spore densities of *P. tomentosa* and *P. nigra*. Previous studies have shown that there are differences in AMF diversity and community structure among sunflower varieties and genotypes (Turrini et al., 2016), as well as among papaya varieties (Khade et al., 2010). There were differences in AMF of poplar roots among tree species; the richness and diversity of *P. nigra* in autumn were significantly higher than in other tree species, and the number of unique ASVs was greatest. Beta diversity analysis showed that the three tree species had different AMF community compositions. These different compositions may be related to the structural differences of the varieties (e.g., root diameter, length, and density), which affect soil nutrient uptake, as well as the community composition and root symbiosis functions of AMF (Baon et al., 1994; Maherali, 2014). In our experiment, *P. nigra* had more tender, fibrous roots. In previous studies, Lingua et al. found that *P. alba* and *P. nigra* inoculated with AMF were suitable for phytoremediation under Zn stress, whereas *P. alba* performed better when inoculated with *Glomus mosseae* (Lingua et al., 2008). Salehi et al. found that *Populus deltoides* showed higher tolerance to Pb stress, and *P. tomentosa* inoculated with AMF increased its biomass under Pb stress (Salehi et al., 2014). In this study, we found that the host plant was able to significantly affect the distribution of AMF. Varieties of poplars had different abilities to recruit AMF. Compared with *P. simonii*, *P. tomentosa* and *P. nigra* had a closer symbiotic relationship with AMF. These results provide a reference for the selection of tree species in future afforestation, and for analyses of the relationships between AMF and different poplar varieties.

GRSP can be used as a biological indicator of forest soil pollution (Wang et al., 2020). An investigation of humid forests in southern Cameroon showed that GRSP is important for the accumulation of soil carbon and nitrogen (Fokom et al., 2012). Our study also showed the significance of GRSP with respect to soil N accumulation, highlighting the positive effects of soil aggregate stability on nitrogen accumulation (Zhu et al., 2018). Jahantigh et al. found that the addition of potassium and mycorrhizal fungi can increase the yield of GRSP and enhance plant resistance to heavy metal stress (Jahantigh et al., 2022). We also found a positive correlation between K and EEG content. The colonization intensity of mycorrhiza was positively correlated with soil P, K, EC, N, and AK. In a previous investigation of *Acaena elongata*, colonization intensity was positively correlated with K and pH (Vázquez-Santos et al., 2019), consistent with our findings. Mycorrhizal colonization increases the accumulation of N in soil and N absorption by the host plant, but Egerton-Warburton and Allen showed that high nitrogen reduces mycorrhizal colonization (Egerton-Warburton and Allen, 2000). Although we found the opposite interaction, these findings suggest that the relationship between AMF and soil N content is sensitive; it may be altered by changes in altitude, temperature, precipitation, soil type, and host plant. In the present study, AMF spore density was negatively correlated with SOM, which was similar to the results of previous studies in the Universitas Sebelas Maret educational forest (Dewi et al., 2021) and a Chilean Mediterranean-type ecosystem (Silva-Flores et al., 2019); thus, low SOM is presumably associated with high mycorrhizal spore density. Multiple variables may have some effects on the composition of the AMF community. The richness and diversity of the AMF community will increase with increasing K and pH but decrease under high levels of NH_4_^+^·N. The increase in P will also improve the community richness; the increase in NO_3_^−^·N will increase the diversity. Potassium ion concentration and soil acid–base properties may have important roles in the construction of the AMF community. Overall, soil K had the greatest impact on the AMF community. Potassium could directly affect the production of glomalin in soil and the degree of mycorrhizal colonization; it also had robust predictive ability with respect to mycorrhizal diversity and richness. Previous studies showed that the soil exchangeable potassium concentration had an important effect on the AMF community structure in the rhizosphere of *Populus-Salix* in the riparian zone (Öpik et al., 2006). There is evidence that soil pH is an important environmental factor that can predict the AMF community and a primary factor that can control the AMF niche space (Dumbrell et al., 2010; Van Geel et al., 2018). Our investigation showed that soil pH had a predictive role with respect to AMF community richness. In several studies concerning large numbers of soil samples collected from natural ecosystems worldwide, the AMF Virtual Taxonomic Group has shown that pH is the most important abiotic driver; moreover, the relative abundances of virtual taxa are driven by environmental variables (Davison et al., 2021).

In this study, soil pH, K, AK, N, and P were important environmental factors that affected the AMF community composition, indicating that soil physical and chemical properties play important roles in regulating the AMF community. Soil physical and chemical properties can directly affect EEG, thus influencing the AMF community. Soil EEG represents the AMF community richness in host plant roots, and the structural equation model showed that EEG content had positive effects on richness and diversity. Additionally, a relationship was predicted between spore density and community richness, implying that spore density was a good index for detecting AMF community richness in roots.

## Conclusions

This study revealed the diversity of AMF communities in poplar roots and its relationship with environmental factors. The AMF community was richer in autumn than in spring; niches and opportunities were broader in autumn. Furthermore, this study revealed differences in the sensitivity of AMF communities to seasons, as well as correlations with host age and species. These environmental factors and soil properties affected the formation of the poplar–AMF symbiotic system. The presence of water, temperature needed for photosynthesis, and exposure are important factors regulating physiological plant status and symbiotic relationships. The sensitivity of AMF to soil pH and potassium corresponded to changes in their communities. The results enhance our understanding of the mechanism underlying the relationship between soil nutrients and the soil–AMF community, especially in the context of poplar afforestation. Considering the important contribution of glomalin to soil aggregation, our study showed that glomalin content was closely related to the AMF community, which has potential implications for measuring AMF community function. The sensitivity of the AMF community was closely related to the growth of host plants, which will also aid prediction of mycorrhizal changes of plants at different stages in complex forest ecosystems.

## Data availability statement

The datasets presented in this study can be found in online repositories. The names of the repository and accession number can be found below: NCBI; PRJNA880710.

## Author contributions

SH wrote the manuscript and analyzed the data. XH and GZ conceived the idea of this study. XW and GW processed the experimental samples. SH and YC completed data collection. XYD participated the figure drawing all of the authors contributed to its further improvement. All authors contributed to the article and approved the submitted version.
