# Supplementary material for: Multidimensional analysis reveals environmental factors that affect community dynamics of arbuscular mycorrhizal fungi in poplar roots
Source: Front Plant Sci. 2023 Jan 17;13:1068527. doi: 10.3389/fpls.2022.1068527 (PMC9887326; doi:10.3389/fpls.2022.1068527)
Supplement: Supplementary file 1 [file DataSheet_1.docx]

Supplementary Material

# Supplementary Figures and Tables

## Supplementary Figures

**
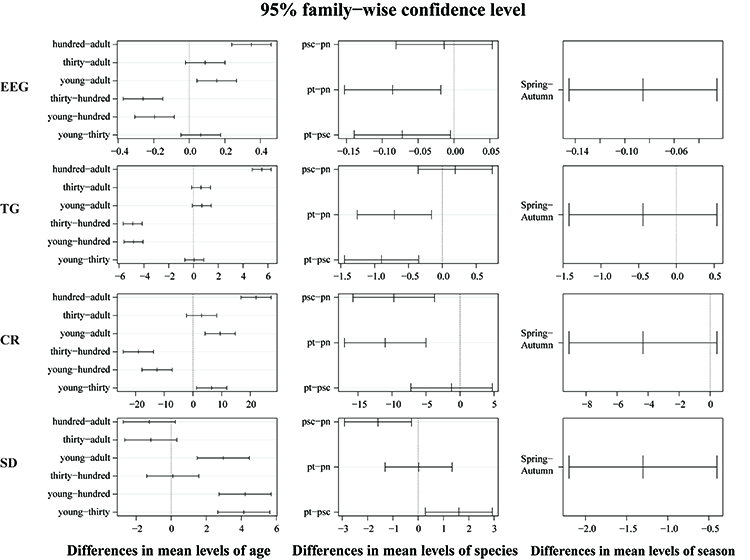
**

**Fig S1.** Tukey HSD analysis of age, tree species and seasonal factors. p<0.05

**
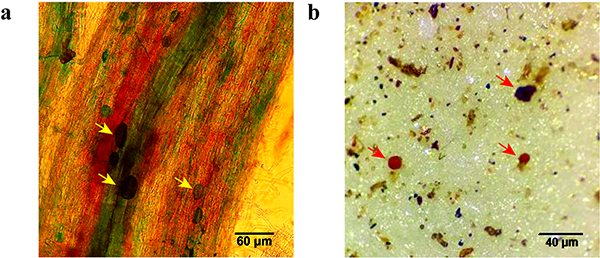
**

**Fig S2.** AMF colonization of poplars. a. Mycorrhiza microstructure (arrows: vesicles). b. AMF spores in soil (arrows).

**
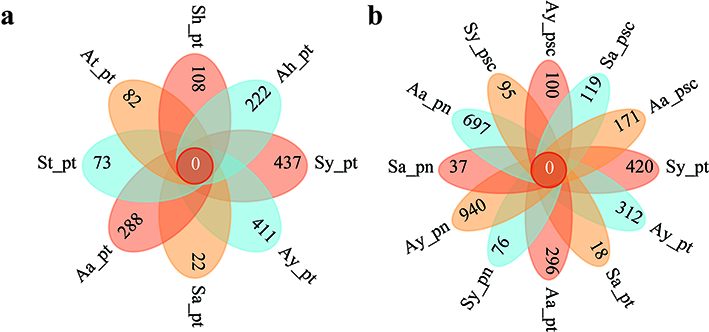
**

**Fig S3.** VENN diagram of AMF community based on ASV level for poplar root system in two seasons. a Different tree ages. b. Different tree species. The uppercase letter S stands for spring and A stands for autumn. After the capital letter, the lowercase letter y stands for youth, a stands for adulthood, t stands for thirty years, and h stands for a hundred years.

**
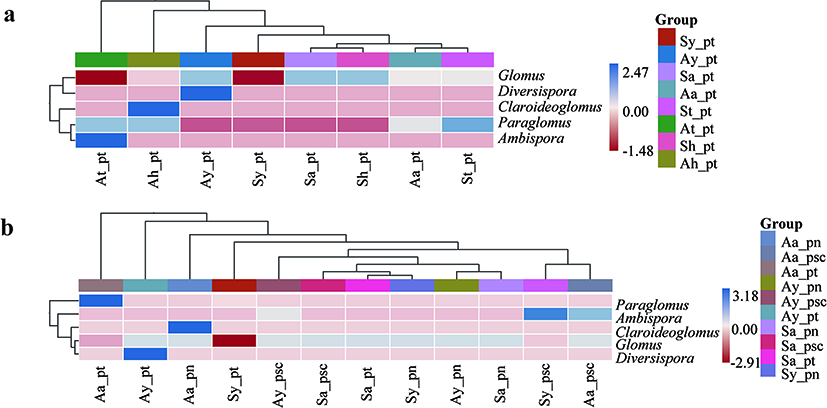
**

**Fig S4.** Heat map of species composition at genus level of AMF in two seasons. a. Different tree ages. b. Different tree species. The uppercase letter S stands for spring and A stands for autumn. After the capital letter, the lowercase letter y stands for youth, a stands for adulthood, t stands for thirty years, and h stands for a hundred years.

**
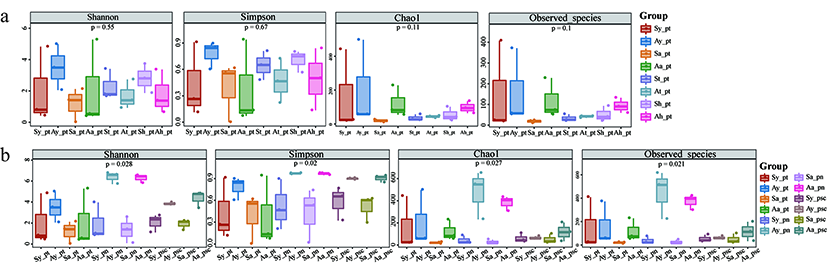
**

**Fig S5.** Diversity of AMF in poplar roots. α diversity index of AMF community of poplar roots in two seasons. a. Different tree ages. b. Different tree species. The points jittered around the boxplot represent the raw data. The significance of difference was tested by dunn’test (**, p < 0.01;***,p<0.001).

**
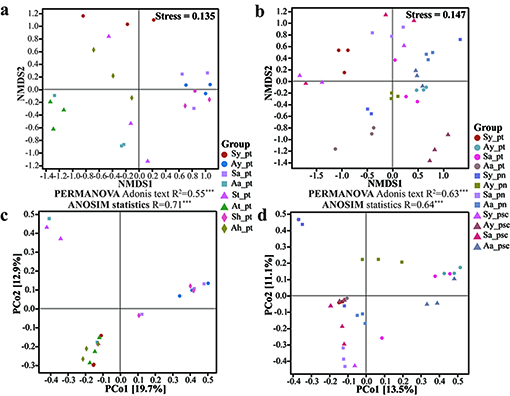
**

**Fig S6.** β diversity analysis of AMF community based on Bray_Curtis distance poplar roots in two seasons. a & c. Different tree ages. b & d. Different tree species.

**
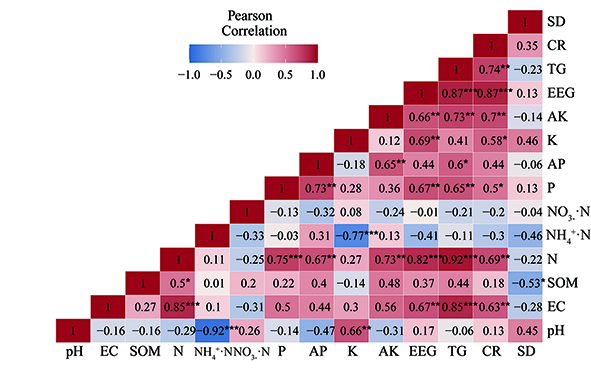
**

**Fig S7.** Heat map for correlation analysis of environmental factors in poplar rhizosphere. *, 0.01 < p < 0.05; **, 0.001 < p < 0.01; ***, p < 0.001.

**
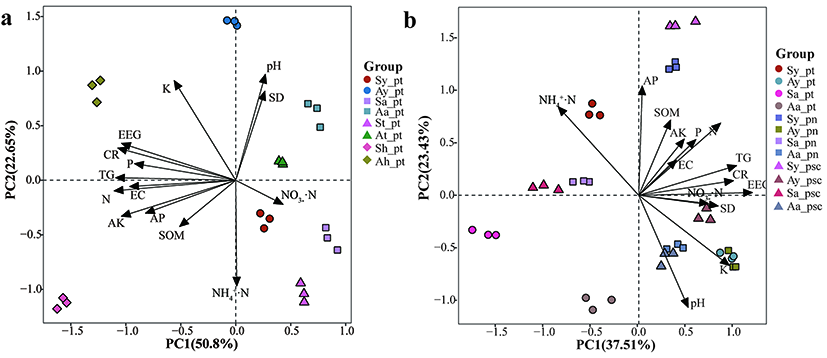
**

**Fig S8.** PCA analysis of rhizosphere environmental factors of poplars in two seasons. a. Different tree ages. b. Different tree species.

## Supplementary Tables

**Table S1.** Soil parameters of poplar rhizosphere in two seasons.

| Soil parameters | | pH | EC(μs·cm^-1^) | SOM(g·kg^-1^) | N(mg·kg^-1^) | NH_4_^+^·N(mg·kg^-1^) | NO_3_^-^·N(mg·kg^-1^) | P(mg·kg^-1^) | AP(mg·kg^-1^) | K(%) | AK(mg·kg^-1^) |
| --- | --- | --- | --- | --- | --- | --- | --- | --- | --- | --- | --- |
| Spring | y_pt | 7.55±0.05g | 99.23±1.34g | 6.49±0.03h | 385.33±6.51gh | 4.18±0.04d | 3.88±0.04h | 750.67±9.71c | 32.13±0.55b | 1.6±0.03f | 103±2f |
|  | a_pt | 7.79±0.04f | 112.67±0.78e | 3.91±0.04j | 210.33±1.53j | 4.87±0.04a | 1.31±0.03m | 397.33±3.51j | 6.92±0.1g | 1.59±0.02f | 82.3±0.82h |
|  | t_pt | 7.41±0.04hi | 114.23±0.06d | 8.04±0.06e | 389±4.58g | 4.91±0.05a | 8.03±0.04b | 394±9.17j | 3.12±0.11k | 1.61±0.02ef | 131.67±3.51d |
|  | h_pt | 7.38±0.02i | 134.93±2.21b | 27.13±0.45a | 1246.67±20.82a | 4.57±0.05b | 2.92±0.04k | 837.67±16.8a | 33.73±0.65a | 1.67±0.02de | 330±3a |
|  | y_pn | 7.45±0.02h | 107±2.02f | 9.92±0.03c | 489.33±9.02e | 3.85±0.04f | 3.56±0.05i | 512±5.57h | 25.17±0.38c | 1.68±0.01de | 318.67±3.21b |
|  | a_pn | 7.61±0.04g | 109.97±1.99ef | 7.41±0.02f | 382.33±14.57gh | 4.55±0.04b | 3.25±0.03j | 435.67±6.81i | 6.07±0.05h | 1.65±0.02e | 107.33±2.52ef |
|  | y_psc | 7.42±0.02h | 120.83±1.2c | 12.37±0.25b | 676.33±6.03c | 4.08±0.04e | 6.73±0.04e | 796.67±8.08b | 24.17±0.4d | 1.63±0.01ef | 103.67±2.52f |
|  | a_psc | 7.42±0.01h | 104.93±1.53fg | 7.07±0.04g | 357.67±6.51i | 4.38±0.03c | 2.89±0.03k | 452±9.17i | 3.86±0.06j | 1.66±0.01de | 86.73±0.8g |
| Autumn | y_pt | 8.1±0.02c | 113.13±3.5de | 3.57±0.15k | 619.33±15.57d | 0.92±0.07k | 3.25±0.07j | 681±11.53d | 5.43±0.08i | 1.89±0.03a | 111.33±3.06e |
|  | a_pt | 8.21±0.03a | 98.1±3.32g | 5.74±0.09i | 202.67±4.51k | 0.67±0.04l | 4.67±0.04g | 518±6.56gh | 6.8±0.07g | 1.68±0.03de | 83.17±0.78h |
|  | t_pt | 8.14±0.01b | 104.4±1.11fg | 26.07±0.81a | 356.33±10.07i | 0.38±0.04m | 7.35±0.04c | 406±8.89j | 9.95±0.06e | 1.7±0.02d | 128±2d |
|  | h_pt | 8±0.02e | 143.63±3.12a | 8.2±0.05d | 929.67±7.09b | 1.26±0.05i | 2.3±0.11l | 753.33±30.55bc | 25.15±0.57cd | 1.9±0.04a | 230.33±54.88c |
|  | y_pn | 8.15±0.03ab | 113.17±1.89de | 5.62±0.34i | 434.67±7.57f | 0.36±0.04m | 4.87±0.06f | 529.67±4.51g | 6.15±0.07h | 1.82±0.03b | 145±2.65c |
|  | a_pn | 8.17±0.02ab | 111.87±1.5e | 10.18±0.17c | 373±7h | 1.72±0.04g | 7.05±0.07d | 550.67±5.13f | 1.94±0.08l | 1.77±0.02bc | 103.33±2.08f |
|  | y_psc | 8.11±0.03bc | 113.03±2.76de | 7.09±0.2fg | 481±4e | 1.55±0.03h | 7.45±0.09c | 531±6g | 8.82±0.06f | 1.75±0.02c | 155.33±3.51c |
|  | a_psc | 8.05±0.01d | 99.57±3.76g | 10.03±0.16c | 339.33±13.58i | 1.16±0.04j | 8.87±0.08a | 627.67±4.73e | 5.45±0.04i | 1.82±0.02b | 108±2ef |

The lowercase letter y stands for youth, a stands for adulthood, t stands for thirty years, and h stands for a hundred years. In the treatment of horizontal mark comparison, the significance of difference was tested by Student's t-test. Different letters indicate that there are significant differences at the p=0.05 probability level. All data were mean ± standard error (Mean ± SE).

**Table S2.** Analysis of Variance of age, tree species and seasonal factors of soil parameters.

| Soil parameters | | Df | Sum sq | Mean sq | F value | Pr (>F) |
| --- | --- | --- | --- | --- | --- | --- |
| pH | Age | 3 | 0.3054 | 0.1018 | 0.972 | 0.425 |
|  | Species | 2 | 0.156 | 0.07811 | 0.77 | 0.471 |
|  | Season | 1 | 4.477 | 4.477 | 399.2 | <2e-16** |
| EC | Age | 3 | 4753 | 1584.4 | 33.37 | 5.59e-08** |
|  | Species | 2 | 150.3 | 75.15 | 1.564 | 0.224 |
|  | Season | 1 | 9 | 8.93 | 0.059 | 0.809 |
| SOM | Age | 3 | 929 | 309.67 | 5.929 | 0.00459** |
|  | Species | 2 | 119.0 | 59.52 | 16.38 | 1.15e-05*** |
|  | Season | 1 | 6.4 | 6.41 | 0.137 | 0.712 |
| N | Age | 3 | 2646629 | 882210 | 74.64 | 4.89e-11*** |
|  | Species | 2 | 72443 | 36222 | 2.028 | 0.148 |
|  | Season | 1 | 3015 | 30150 | 0.429 | 0.516 |
| NH_4_^+^·N | Age | 3 | 0.45 | 0.149 | 0.033 | 0.992 |
|  | Species | 2 | 0.2 | 0.0992 | 0.032 | 0.968 |
|  | Season | 1 | 140.6 | 140.60 | 761.1 | <2e-16*** |
| NO_3_^-^·N | Age | 3 | 99.52 | 33.17 | 35.22 | 3.57e-08*** |
|  | Species | 2 | 62.05 | 31.024 | 9.841 | 0.000445*** |
|  | Season | 1 | 32.79 | 32.79 | 7.079 | 0.0107* |
| P | Age | 3 | 669937 | 223312 | 103.1 | 2.46e-12*** |
|  | Species | 2 | 62324 | 31162 | 2.279 | 0.118 |
|  | Season | 1 | 85 | 85 | 0.004 | 0.95 |
| AP | Age | 3 | 2161 | 720.4 | 11.51 | 0.000133*** |
|  | Species | 2 | 58 | 23.13 | 0.296 | 0.746 |
|  | Season | 1 | 804 | 803.8 | 7.881 | 0.0073** |
| K | Age | 3 | 0.09141 | 0.03047 | 2.555 | 0.0842 |
|  | Species | 2 | 0.01115 | 0.005575 | 0.6 | 0.555 |
|  | Season | 1 | 0.2868 | 0.28675 | 74.04 | 3.86e-11*** |
| AK | Age | 3 | 142257 | 47419 | 44.88 | 4.55e-09*** |
|  | Species | 2 | 35220 | 17610 | 5.638 | 0.00783** |
|  | Season | 1 | 7415 | 7415 | 1.234 | 0.272 |

ns, no significant;*,0.01<p<0.05;**,0.001<p<0.01;***, p<0.001

**Table S3.** Analysis of Variance of age, tree species, and season factors.

| Variables | | Df | Sum sq | Mean Sq | F value | Pr (>F) |
| --- | --- | --- | --- | --- | --- | --- |
| EEG | Age | 3 | 0.3953 | 0.13175 | 27.92 | 2.38e-07*** |
|  | Species | 2 | 0.05086 | 0.025430 | 3.574 | 0.0393* |
|  | Season | 1 | 0.0872 | 0.08722 | 8.225 | 0.00622** |
| TG | Age | 3 | 117.66 | 39.22 | 176.2 | 1.51e-14*** |
|  | Species | 2 | 5.373 | 2.6863 | 7.247 | 0.00246** |
|  | Season | 1 | 2.35 | 2.349 | 0.827 | 0.368 |
| CR | Age | 3 | 1714.1 | 571.4 | 53.95 | 9.1e-10*** |
|  | Species | 2 | 870.5 | 435.2 | 12.14 | 0.000112*** |
|  | Season | 1 | 225.3 | 225.33 | 3.35 | 0.0737 |
| SD | Age | 3 | 69.89 | 23.298 | 27.6 | 2.61e-07*** |
|  | Species | 2 | 20.48 | 10.241 | 5.86 | 0.00664** |
|  | Season | 1 | 20.28 | 20.28 | 8.452 | 0.00559** |

EEG: Easily extract glomalin-related soil protein, TG: Total glomus-related protein; CR: colonization rate, SD: spore density

ns, no significant;*,0.01<p<0.05;**,0.001<p<0.01;***, p<0.001
